# The Effects of Suprabullar Pneumatization on the Orientation of Its Surrounding Anatomical Structures Relevant to the Frontal Drainage Pathway

**DOI:** 10.3390/diagnostics12010052

**Published:** 2021-12-27

**Authors:** Nikma Fadlati Umar, Mohd Ezane Aziz, Norhafiza Mat Lazim, Baharudin Abdullah

**Affiliations:** 1 Department of Otorhinolaryngology-Head & Neck Surgery, School of Medical Sciences, Universiti Sains Malaysia, Kubang Kerian 16150, Kelantan, Malaysia; nikmafadlati81@gmail.com (N.F.U.); norhafiza@usm.my (N.M.L.); 2 Department of Radiology, School of Medical Sciences, Universiti Sains Malaysia, Kubang Kerian 16150, Kelantan, Malaysia; drezane@usm.my

**Keywords:** suprabullar pneumatization, suprabullar cell, frontal recess, anterior ethmoidal artery, middle ethmoidal artery, posterior ethmoidal artery

## Abstract

Objective: The aim of this study was to evaluate the effects of suprabullar pneumatization on the orientation of the frontal sinus outflow structures and its association with the volume of anterior ethmoid sinus. Methods: A retrospective chart review of computed tomography of paranasal sinuses (CTPNS) images was conducted. A total of 370 sides of the CTPNS of 185 patients were analyzed. Results: The course of anterior ethmoidal artery (AEA) along the skull base (*p* = 0.04) and position of AEA at the second lamella (*p* = 0.04) was significantly associated with the type of suprabullar pneumatization. The AEA is expected to be lower at the skull base and at a longer distance from the second lamella with the increase in grading of the suprabullar pneumatization. The distance of AEA to the second lamella (*p* < 0.001) and third lamella (*p* = 0.04) was significantly different depending on the type of suprabullar pneumatization, which indicates AEA is expected to be at a longer distance from the second lamella and third lamella in higher grade suprabullar pneumatization. The type of suprabullar pneumatization has a significant but weak association with the anterior ethmoid sinus volume (*p* = 0.04). Conclusions: There is a significant effect of the type of suprabullar pneumatization on the orientation of the surrounding anatomical structures at the frontal recess. The type of suprabullar pneumatization is influenced by the anterior ethmoid sinus volume, which suggests it has a possible role in the frontal drainage pathway.

## 1. Introduction

**Figure 1:**
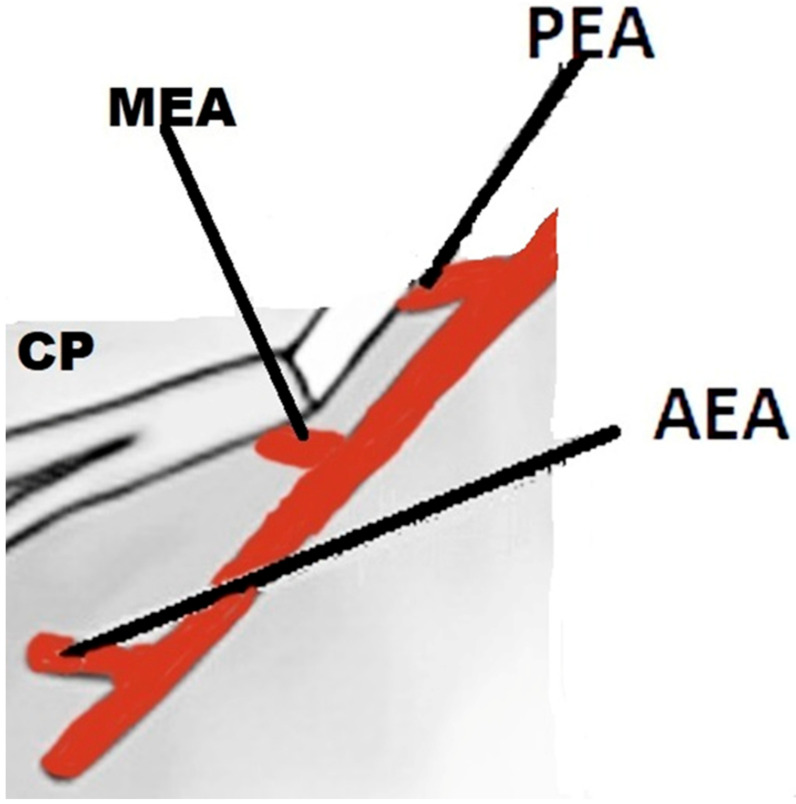
Relation of the ethmoidal arteries at the left cribriform plate to the anterior. Ethmoidal artery (AEA) running in front and the posterior ethmoidal artery (PEA) at the back. Middle ethmoidal artery (MEA), when present, is located between the anterior and posterior ethmoidal arteries. AEA, anterior ethmoidal artery; MEA, middle ethmoidal artery; PEA, posterior ethmoidal artery; and CP, cribriform plate.

It is vital to have sound anatomical knowledge and recognize anatomical variations in each patient prior to endoscopic sinus surgery (ESS) [1]. In order to improve the surgical outcome, it is imperative during ESS to eradicate ethmoid and frontal diseases and facilitate drainage of the frontal sinus. Inadequate clearance of disease may necessitate revision surgery with a higher risk of complications. One of the most feared complications while dissecting around the frontal recess is an iatrogenic injury of 0.3% to the anterior ethmoidal artery (AEA), leading to retro-orbital hematoma, vision loss and cerebrospinal fluid leak [2]. The AEA exits the canal above the cribriform plate and enters the nasal cavity via the anterior ethmoidal orifice of the cribriform plate, dividing into anterior septal and anterior lateral nasal branches. Its terminal branch is the external nasal artery [2,3]. Inadvertent injuries to AEA could occur during dissection along the frontal recess. The frontal recess opening has a variable position that may result in failure to identify the important structures around it. The uncertainty and confusion arise due to the existence of other air cells in close relation to the frontal sinus opening. In addition, the variable presence of middle ethmoidal artery (MEA) continues to be the subject of debate. MEA enters the nasal cavity through a foramen at the ethmoid bone in between the anterior and posterior ethmoidal arteries (Figure 1). The realization of a third ethmoidal artery coexisting with the anterior and posterior ethmoidal arteries followed the discovery of accessory ethmoidal vessels. Two cadaveric studies confirmed the presence of an accessory ethmoidal foramina that contained the third ethmoidal artery [4,5]. This artery was initially named the ‘tertiary’ ethmoidal artery.

The pattern of pneumatization of suprabullar cells has been described as a system to identify the frontal recess and AEA [6]. By using computed tomography of paranasal sinus (CTPNS), the relation of the two structures with the suprabullar cell could be identified and helps to avoid complications. International frontal sinus anatomy classification (IFAC) defines suprabullar cell as a posterior cell that pushes the frontal drainage pathway anteriorly [7]. Although IFAC does not include suprabullar pneumatization in its classification, understanding its significance on the surrounding anatomical structures could serve as an additional assessment besides IFAC. The suprabullar cells, together with the agger nasi, frontoethmoid, ethmoid bulla, supraorbital and frontal bullar cells, are related with the frontal sinus outflow tract [8]. The suprabullar cells are found above the ethmoid bulla and behind the second lamella. The classification of suprabullar pneumatization is divided into four types based on the number and position of air cells in relation to the ethmoid bulla, suprabullar space and frontal sinus [6]. It has been demonstrated that the classification of the suprabullar pneumatization is expedient to identify the AEA and frontal recess [6]. In addition to the number of air cells, the volume anterior ethmoid sinus may also influence their relationship with the surrounding structures. The aim of this study was to evaluate (i) the effects of suprabullar pneumatization on the orientation of the frontal sinus outflow structures; and (ii) the association between the volume of anterior ethmoid sinus and the type of the suprabullar pneumatization. 

## 2. Materials and Methods

The study protocol was reviewed and approved by the research ethics committee (USM/JEPeM/18120164) and was performed in adherence with the Declaration of Helsinki. A retrospective chart review of CTPNS images from the Department of Radiology of a tertiary hospital from January 2010 to December 2018 was conducted. The CTPNS images were selected randomly based on the inclusion criteria of subjects aged 18 years and above, along with CT images with a slice thickness of 1 mm and capable of multiplanar reconstruction (MPR). The subjects were patients without any sinonasal conditions who underwent CT examination as part of the investigations for their presenting illness. The exclusion criteria were previous surgery to paranasal sinuses and skull base, skull base or facial trauma, and craniofacial abnormality. The CTPNS images were retrieved from Radiology Information System (RIS) and Picture Archive Communication System (PACS). These CTPNS images were acquired from Siemens SOMATOM Definition AS+, which can produce 128 slices of images per rotation. Multiplanar CTPNS images were reconstructed to axial, coronal and sagittal view, at 1 mm slice thickness. The CTPNS images were interpreted by 1 radiologist (M.E.A.) and 2 otorhinolaryngologists (B.A., N.F.U.). The CT examiners evaluated all measurements independently from each other. When there was no 100% consensus, the evaluations were resolved by consensus in the presence of another otorhinolaryngologist (N.M.L.) familiar with the frontal sinus anatomy. All data obtained were entered in the study proforma.

### 2.1. Evaluation of Suprabullar Pneumatization and Anterior Ethmoid Sinus Volume

**Figure 2:**
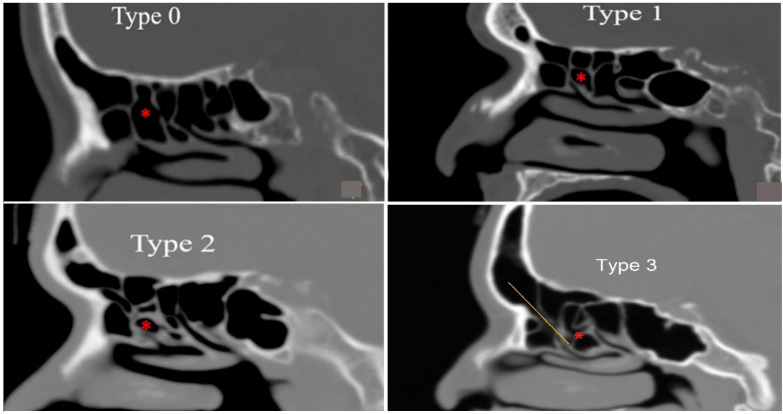
The suprabullar pneumatization is divided into four types based on the number and position of air cells in relation to the ethmoid bulla (red asterisk). Type 0 is absence of suprabullar air cells between ethmoid bulla and skull base, Type 1 is presence of a single suprabullar air cell between ethmoid bulla and skull base, Type 2 is presence of multiple suprabullar air cells above the ethmoid bulla without extension into the frontal sinus and Type 3 suprabullar cell is extension of suprabullar air cell into the frontal sinus displacing the frontal sinus drainage anteriorly (yellow line). Scale bar, 1 cm is equal to 5 cm.

A total of 370 sides of the CTPNS of 185 patients were analyzed, consisting of 86 (46.4%) men and 99 (53.5%) women with age between 18 and 84 years old (mean age 46.8). The type of suprabullar pneumatization was determined by sagittal CTPNS to identify the attachment of the second lamella to the skull base. It was classified according to Tan et al. [6] as type 0, type 1, type 2 and type 3 (Figure 2):

- Type 0 was absent of suprabullar air cells between ethmoid bulla and skull base;
- Type 1 was presence of a single suprabullar air cell between ethmoid bulla and skull base;
- Type 2 was presence of multiple suprabullar air cells above the ethmoid bulla, without extension into the frontal sinus;
- Type 3 was presence of either a single or multiple suprabullar air cells above the ethmoid bulla, with extension into the frontal sinus;

Anterior ethmoid sinus volume was calculated by combining the volume of the ethmoid bulla and suprabullar cells based on a validated method for ethmoid sinus volume assessment [9]. The volume was defined as ‘the longest anteroposterior diameter in the parasagittal plane’ times ‘the longest vertical diameter in the parasagittal plane’ times ‘the longest diameter from side to side in the coronal plane’ of the ethmoid bullar and suprabullar cells. The calculated volume was measured in cm^3^.

### 2.2. Evaluation of Anterior Ethmoidal and Middle Ethmoidal Arteries

**Figure 3:**
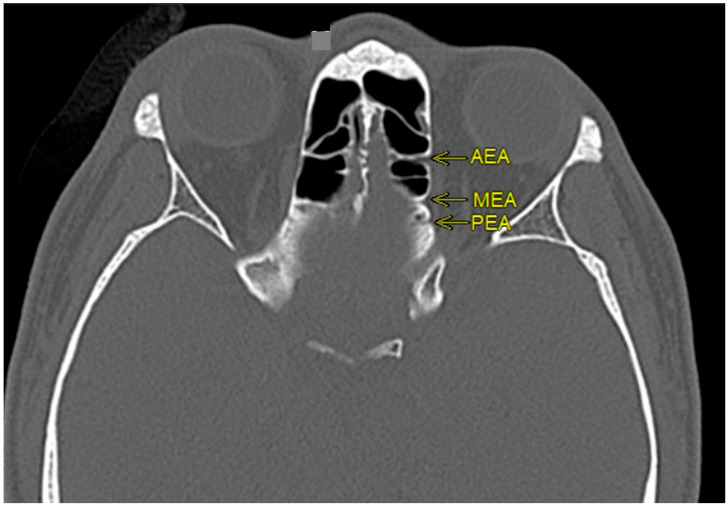
Computed tomography of paranasal sinuses (axial view) of the anterior ethmoidal (AEA), middle ethmoidal (MEA) and posterior ethmoidal arteries (PEA).

**Figure 4:**
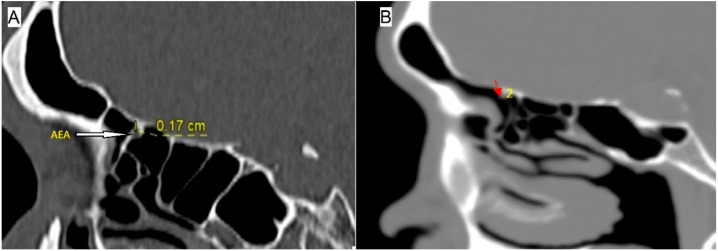
Location of anterior ethmoidal artery (white arrow) at the second lamella (**A**). The distance of anterior ethmoidal artery from the skull base is measured in a vertical line, and 6.2% of anterior ethmoidal artery (red arrow) was identified between the first and second lamellae (2) (**B**).

The identification of AEA, MEA and posterior ethmoidal artery (PEA) was initially made by coronal CTPNS and subsequently confirmed with axial and sagittal plane (Figure 3). Once identified, the position of these arteries was established by the sagittal view. The measurements were made by zooming in the image using the sagittal section. The position of AEA at the lamella was assessed in the locations it had been previously identified [10,11]; between 1st and 2nd lamellae, within posterior margin of frontal sinus ostium, within 2nd lamella, between 2nd and 3rd lamellae, within 3rd lamella and posterior to 3rd lamella. The position of MEA at the lamella was assessed anterior to 3rd lamella, within 3rd lamella and between 3rd lamella and anterior wall of the sphenoid sinus. The course of the ethmoidal arteries at the skull base was measured by their vertical distance to the skull base (Figure 4). There are three known types of relation of ethmoidal arteries to the skull base; Type 0 is when the artery runs in a bony canal, Type 1 is when the artery runs in a short mesentery at a vertical distance of less than 1 mm to the skull base and Type 2 is when the artery runs in a long mesentery at a vertical distance of 1 mm or more to the skull base [12].

### 2.3. Measurement of Anterior Ethmoidal and Middle Ethmoidal Arteries to Related Anatomical Structures

The measurements of the distances from AEA to the nasofrontal beak, AEA to posterior boundary of frontal sinus, AEA to second lamella and AEA to third lamella were done using the sagittal section of CTPNS. Initially, the first vertical line was made across the AEA and the second vertical line was made across the chosen structure individually. Then, the measurement was performed in the transverse plane between the first vertical and second vertical lines. The measurements of the distance from AEA to MEA, MEA to PEA and AEA to PEA were done in similar manner. 

### 2.4. Statistical Analysis

All data obtained were transferred into the Statistical Package for Social Sciences (SPSS) software version 22.0. Descriptive analysis was used to calculate the prevalence of each type of suprabullar pneumatization, the type and position of AEA and MEA relative to the skull base, and the distance of the AEA to the related anatomical structures. The association of type of the suprabullar pneumatization with the sociodemographic and characteristics of participants was determined by Chi-Square test. Pearson Chi-Square test was used to determine the association between the type of suprabullar pneumatization with course of AEA along the skull base and position of AEA at the second lamella. The ANOVA test [13] was used for the association between each type of suprabullar pneumatization and the distance of AEA to the nasofrontal beak, distance of AEA to posterior boundary of frontal sinus, distance of AEA to second lamella, distance of AEA to third lamella, distance of AEA to MEA and distance of AEA to PEA. Likewise, the association between anterior ethmoid sinus volume and the type of suprabullar pneumatization was made by ANOVA test. The correlation between the distance of AEA and the surrounding anatomy to the anterior ethmoid sinus volume was assessed by Pearson correlation test. The correlation was considered weak when r < 0.4, moderate at 0.4 to 0.7, strong at 0.7 to 0.9 and very strong > 0.9 [13]. Post hoc analysis using bonferroni correction was performed for the correlation between suprabullar pneumatization and ethmoidal arteries and between suprabullar pneumatization and anterior ethmoid sinus volume [14]. A *p*-value of < 0.05 was considered statistically significant.

## 3. Results

### 3.1. Demographic Data

The most prevalent type of suprabullar pneumatization was type 2 (45.9%), followed by type 1 (22.7%), type 3 (17.8%) and type 0 (13.5%). There was no association between the type of suprabullar pneumatization and age (*p* = 0.36), gender (*p* = 0.15) and ethnicity (*p* = 0.21) (Table 1).

### 3.2. Suprabullar Pneumatization and Ethmoidal Arteries

The presence of AEA was identified in 100%, MEA in 14.1% and PEA in 97% of CTPNS samples. At the skull base, 54.3% of AEA coursed in a long mesentery (type 2), 27.3% in a short mesentery (type 1) and 18.4% in a bony canal (type 0). Along the skull base, 44.2% of MEA coursed in a long mesentery (type 2), 36.6% in a bony canal (type 0) and 19.2% in a short mesentery (type 1). The position of AEA was identified between 2nd and 3rd lamellae in 60.5%, 16.5% at 2nd lamella, 13.5% at 3rd lamella, 6.2% between 1st and 2nd lamellae, 2.2% within posterior margin of frontal sinus ostium and 1.1% between 3rd lamella and anterior wall of sphenoid. The position of MEA was identified between 3rd lamella and anterior wall of sphenoid in 82%, 15.4% at 3rd lamella and 1.92% between 2nd and 3rd lamellae. The course of AEA along the skull base (*p* = 0.04) and position of AEA at the second lamella (*p* = 0.04) was significantly associated with the type of suprabullar pneumatization. This association means that the position of AEA is expected to be lower at the skull base and at a longer distance to the second lamella with the increase in grading of the suprabullar pneumatization. In contrast, no association was found between the course of MEA along the skull base (*p* = 0.60) and position of MEA at the third lamella (*p* = 0.36) with the type of suprabullar pneumatization. The relation of AEA to the surrounding anatomical structures is shown in Table 2. The distance of AEA to the second lamella and third lamella was significantly different depending on the type of suprabullar pneumatization with a *p*-value of <0.001 and 0.04, respectively. In other words, the position of AEA is expected to be at a longer distance from the second lamella and third lamella in higher grade, as compared to lower grade suprabullar pneumatization. Based on the post hoc comparison using Bonferroni correction, there was significant difference in terms of distance of AEA to second lamella between type 0 and type 3 (95% CI −0.59, −0.18; *p* < 0.001), type 1 and type 3 (95% CI −0.52, −0.18; *p* < 0.001) and type 2 and type 3 (95% CI −0.42, −0.12; *p* < 0.001). No significant difference of distance of AEA to second lamella was found for the other pairs. There was significant difference of distance of AEA to third lamella between type 2 and 3 (95% CI−0.25, −0.01; *p* = 0.03), but no significant difference was found for the other pairs.

### 3.3. Correlation of Suprabullar Pneumatization with Anterior Ethmoid Sinus Volume

There was a significant association between anterior ethmoid sinus volume and the type of suprabullar pneumatization (*p* = 0.04) (Table 3). Post hoc comparison using Bonferroni correction showed no significant difference between all types of suprabullar pneumatization with the volume. A positive weak correlation was demonstrated between the anterior ethmoid sinus volume and the distance of AEA to the skull base (r = 0.18, *p* < 0.001), and distance of AEA to the frontal nasal beak (r = 0.14, *p* < 0.001) (Table 4). However, the other variables were not significantly correlated to the anterior ethmoid sinus volume. 

## 4. Discussion

Among the notable findings in the present study are the prevalence of AEA, MEA and PEA in comparison to previous studies. Whilst the prevalence of AEA of 100% is comparable to other studies [12,15,16], the prevalence of MEA of 14.1% is lower than the prevalence of 28–30% reported previously [12,15]. Additionally, this study found the majority of MEA runs in a long mesentery below the skull base, unlike a previous study, which demonstrated that it runs in a bony canal [15]. The prevalence of PEA is 97 % identical to the reported prevalence of other studies with a range of 86–100% [12,15]. The population in two studies [12,16] was patients without any sinonasal condition who underwent CT investigation for their disorder, similar to the population involved in the present study. In contrast, another study [15] evaluated the ethmoidal arteries in cadaver heads. The identification of AEA position at the skull base is crucial when performing ESS. Several studies had shown that the pneumatization of the ethmoid sinuses and the existence of supraorbital ethmoid cells influence the position of AEA at the skull base [10,17]. Besides reinforcing its findings, the present study demonstrated the position of AEA in relation to its surrounding structures in the presence of suprabullar pneumatization. 

During endoscopic endonasal ethmoidectomy, it is possible to see the AEA only after the lamella connected to the skull base is removed [1]. It is helpful to ascertain the position of the AEA to the lamella while the lamella is being removed, to prevent inadvertent injury during surgery [18]. Although the majority of AEA could be found mostly between the second and third lamellae [10], its position at the lamella may vary according to the degree of suprabullar pneumatization. It should be noted that the distance of AEA to the second and third lamellae might be significantly lengthier when patients have type 3 suprabullar pneumatization, as compared to the other types.

Efforts have been made to map out the location of the frontal sinus opening utilizing virtual endoscopy, a tool based on an analytic software using three-dimensional reconstruction of CTPNS images [19,20]. By analyzing the images, the authors were able to predict the position of the frontal sinus opening, depending on the configuration of the surrounding structures, including the suprabullar cells. It could be argued that with the introduction of IFAC [7,21,22], the frontal sinus drainage patterns and the relationship of frontal sinus opening and its surrounding structures are better understood. However, the classification does not fully explain the process in determining the location of AEA and frontal sinus opening. Understanding of the effect of suprabullar pneumatization on its surrounding anatomical structures serves to complement the IFAC classification. 

One of the most dreaded complications of frontal sinus surgery is injury to the neurovascular structures, particularly the AEA, which can potentially cause blindness. During endoscopic sinus surgery to treat inflammatory nasal polyps associated with chronic rhinosinusitis, the limited confined space of frontoethmoidal area represents a challenge. It is essential to ensure sufficient dissection has been performed with adequate opening of the frontal sinus drainage for ventilation of the frontal sinus. On the other hand, the potential for causing complications is significant. The margin of error in this limited space is small. To avoid complications, the size of the dissecting microinstrument can be selected according to the type of suprabullar pneumatization. A smaller size instrument is perhaps obligatory in the presence of a lower-grade suprabullar pneumatization. Moreover, conditions such as frontal mucocele or sinonasal tumour may require endoscopic draf 2 or 3 surgical approaches. Such surgical procedures require expertise and sufficient anatomical knowledge of the frontoethmoidal area for safer dissection to avoid inadvertent injury to nearby important structures. By understanding the association between the different type of suprabullar pneumatization and the position and course of ethmoidal vessels relative to the surrounding structures, surgeons are able to predict and anticipate the high-risk area where they should be more meticulous and careful in their dissections.

Previous studies found the anterior ethmoid sinus volume ranged between 4.3 cm^3^ and 5.9 cm^3^ [23,24]. The two studies involved Turkish [23] and Romanian [24] subjects, without any sinonasal pathologies who had underwent CT investigation. To the best of our knowledge, no previous study has shown the effect of the anterior ethmoid sinus volume on the frontal sinus drainage. In the present study, the mean volume of the anterior ethmoid sinus was 2.24 cm^3^. This suggests that our population, comprising Asian ethnicities, tends to have smaller volume of anterior ethmoid than the other populations [23,24]. Nonetheless, we would like to highlight that the different methodology used in those two prior studies might have contributed to the discrepancies, which may not be due to the actual difference between the populations. 

Suprabullar pneumatization is directly related to the combined volume of ethmoid and suprabullar air cells that made up the anterior ethmoid sinus. Individuals with large volume of ethmoid and suprabullar air cells tend to have higher grade suprabullar pneumatization, whereby type 3 suprabullar pneumatization can be found in those with the largest volumes. A prior study by Eren et al. [11] showed pneumatization of the ethmoid cell affects the distant of AEA to the frontal nasal beak. Likewise, the present study demonstrated that anterior ethmoid sinus volume affects the distance of AEA to both the skull base and frontal nasal beak.

In the present study, the time taken for each image was approximately 10 to 30 min depending on the level of difficulty. Much of the time taken was to confirm the course and position of AEA, MEA and PEA in the axial, coronal and sagittal CT views. All the structures were identified without much difficulty, especially for the AEA, which can be traced by spotting the ‘nipple sign’ in the coronal view. The challenging part was to determine the presence of MEA, which can be found only in limited number of subjects. Due to this, there were several instances of conflicting opinions, which were settled by consensus, as mentioned. For future study, we would like to suggest the use of cone-beam tomography. Cone-beam computed tomography is an advanced imaging modality being used successfully as an investigative modality in maxillofacial practice. It has less radiation than conventional CT scan; is highly accurate; and provides a three-dimensional volumetric data in axial, sagittal and coronal planes.

The limitations of our study include the data collation from only a single center (monocentric study), subjects with normal anatomy of paranasal sinuses, and anatomical variability of MEA. We excluded patients with chronic rhinosinusitis as the anatomical structures can be distorted or blurred, which can result in imprecise measurements. This is particularly crucial at the ethmoidal sinus, which is a small confined space. Since only patients with normal anatomy of paranasal sinuses were included, the population did not represent patients with chronic rhinosinusitis. The significant but weak association between suprabullar pneumatization and anterior ethmoid sinus can be explored in future studies, in association with chronic rhinosinusitis. It is documented that anatomical variations can occur in the same population; thus, the prevalence of the suprabullar pneumatization found in our study might not be representative of the general population. Similarly, it should not be seen as representing the Asian population. The effect of suprabullar pneumatization in different populations should be investigated due to the variable anatomical structures.

## 5. Conclusions

There is a significant effect of the type of suprabullar pneumatization on the orientation of the surrounding anatomical structures at the frontal recess. The understanding of the effect of suprabullar pneumatization on its surrounding anatomical structures serves to complement the IFAC classification. The position of AEA is expected to be lower at the skull base and at a longer distance to the second lamella with the increase in grading of the suprabullar pneumatization. The type of suprabullar pneumatization is influenced by the anterior ethmoid sinus volume, which suggests it has a possible role in the frontal drainage pathway. Individuals with a large volume of ethmoid and suprabullar air cells tend to have higher grade suprabullar pneumatization, whereby type 3 suprabullar pneumatization can be found in those with the largest volumes. In addition, the distance of AEA to both the skull base and frontal nasal beak is influenced by the anterior ethmoid sinus volume.

## Figures and Tables

**Table 1:** Distribution of suprabullar pneumatization according to patient characteristics.

Variables	CTPNS Sides *n* (%)	Suprabullar Pneumatization Classification				*p* Value *
		Type 0 *n* (%)	Type 1 *n* (%)	Type 2 *n* (%)	Type 3 *n* (%)	
**Age (years)** **(Mean, SD)**	46.83 (19.8)	46.14 (19.1)	49.46 (19.2)	46.75 (19.9)	44.23 (20.9)	0.3 ^a^
**Gender** Male Female	172 (46.5) 198 (53.5)	20 (11.6) 30 (15.2)	44 (25.6) 40 (20.2)	84 (48.8) 86 (43.4)	24 (14.0) 42 (21.2)	0.9 ^b^
**Ethnicity** Malay Chinese Indian	334 (90.3) 32 (8.7) 4 (1.1)	44 (13.2) 6 (18.8) 0	75 (22.5) 8 (25.0) 1 (25.0)	152 (45.5) 15 (46.9) 3 (75.0)	63 (18.9) 3 (9.4) 0	0.2 ^b^
**Total** **(*n*, %)**	370 (100)	50 (13.5)	84 (22.7)	170 (45.9)	66 (17.8)	

* *p* value < 0.05 is significant, ^a^ one-way ANOVA, and ^b^ Pearson Chi-Square test. CTPNS, computed tomography of paranasal sinus.

**Table 2:** The association of distance of anterior ethmoidal artery to the different related anatomical structures for each type of suprabullar pneumatization.

Variables	Type of Suprabullar Pneumatization	Distance of AEA to Different Related Anatomy			*F*-Stat (*df*)	*p* Value * (ANOVA Test)
		Mean	(95% CI)			
			Lower Bound	Upper Bound		
AEA to frontonasal beak	0 1 2 3	12.72 12.90 13.34 13.46	(11.79, 13.64) (12.23, 13.57) (12.86, 13.82) (12.66, 14.26)		0.89 (3369)	0.44
AEA to frontal ostium	0 1 2 3	11.78 11.69 12.20 12.09	(10.67, 12.89) (10.69, 12.69) (11.60, 12.81) (11.13, 13.04)		0.34 (3361)	0.79
AEA to second lamella	0 1 2 3	6.03 6.41 7.13 9.87	(4.81, 7.25) (5.57, 7.24) (6.55, 7.70) (8.80, 10.93)		12.84 (3324)	<0.001
AEA to third lamella	0 1 2 3	5.26 5.18 5.05 6.36	(4.36, 6.16) (4.34, 5.72) (4.62, 5.49) (5.30, 7.43)		2.81 (3315)	0.04
AEA to MEA	0 1 2 3	8.65 8.35 8.70 7.61	(6.83, 10.46) (6.79, 9.92) (7.73, 9.68) (5.66, 9.56)		0.52 (351)	0.67
AEA to PEA	0 1 2 3	11.73 12.04 11.64 12.04	(10.80, 12.65) (11.28, 12.81) (11.16, 12.12) (11.26, 12.82)		0.42 (3307)	0.73

* *p* value < 0.05 is significant. AEA, anterior ethmoidal artery; MEA, middle ethmoidal artery; and PEA, posterior ethmoidal artery.

**Table 3:** The association between anterior ethmoid sinus volume and type of suprabullar pneumatization.

	Type of Suprabullar Pneumatization				*p* Value * (ANOVA Test)
	0	1	2	3	
**Anterior ethmoid sinus volume, cm^3^** **(mean, SD)**	2.15 (0.93)	2.14 (0.87)	2.24 (0.95)	2.45 (0.71)	0.04

* *p* value < 0.05 is significant.

**Table 4:** The correlation between distance of anterior ethmoidal artery and the surrounding anatomy to the anterior ethmoid sinus volume.

Variables	Correlation Coefficient (r)	*p* Value * (Pearson Correlation Test)
Distance of anterior ethmoidal artery to skull base	0.18	<0.001
Distance of anterior ethmoidal artery to frontal nasal beak	0.14	<0.001
Distance of anterior ethmoidal artery to frontal ostium	0.08	0.11
Distance of anterior ethmoidal artery to second lamella	0.01	0.79
Distance of anterior ethmoidal artery to third lamella	0.06	0.22

* *p* value < 0.05 is significant.

## Data Availability

All data generated and analyzed during this study are included in this published article.
